# Representativeness, Vaccination Uptake, and COVID-19 Clinical Outcomes 2020-2021 in the UK Oxford-Royal College of General Practitioners Research and Surveillance Network: Cohort Profile Summary

**DOI:** 10.2196/39141

**Published:** 2022-12-19

**Authors:** Meredith Leston, William H Elson, Conall Watson, Anissa Lakhani, Carole Aspden, Clare R Bankhead, Ray Borrow, Elizabeth Button, Rachel Byford, Alex J Elliot, Xuejuan Fan, Uy Hoang, Ezra Linley, Jack Macartney, Brian D Nicholson, Cecilia Okusi, Mary Ramsay, Gillian Smith, Sue Smith, Mark Thomas, Dan Todkill, Ruby SM Tsang, William Victor, Alice J Williams, John Williams, Maria Zambon, Gary Howsam, Gayatri Amirthalingam, Jamie Lopez-Bernal, F D Richard Hobbs, Simon de Lusignan

**Affiliations:** 1 Nuffield Department of Primary Care Health Sciences University of Oxford Oxford United Kingdom; 2 Immunisation and Vaccine Preventable Diseases Division UK Health Security Agency Colindale, London United Kingdom; 3 Vaccine Evaluation Unit UK Health Security Agency Manchester Royal Infirmary Manchester United Kingdom; 4 Real-time Syndromic Surveillance Team Field Service UK Health Security Agency Birmingham United Kingdom; 5 Royal College of General Practitioners London United Kingdom; 6 Reference Microbiology UK Health Security Agency Colindale, London United Kingdom

**Keywords:** cohort profile, computerized medical record systems, general practice, influenza, COVID-19, sentinel surveillance, syndromic surveillance, serology, virology, public health, digital surveillance, vaccination, primary care data, health data, cohort, virus, immunology, surveillance, representation, uptake, outcome, hospital, sampling, monitoring

## Abstract

**Background:**

The Oxford-Royal College of General Practitioners (RCGP) Research and Surveillance Centre (RSC) is one of Europe’s oldest sentinel systems, working with the UK Health Security Agency (UKHSA) and its predecessor bodies for 55 years. Its surveillance report now runs twice weekly, supplemented by online observatories. In addition to conducting sentinel surveillance from a nationally representative group of practices, the RSC is now also providing data for syndromic surveillance.

**Objective:**

The aim of this study was to describe the cohort profile at the start of the 2021-2022 surveillance season and recent changes to our surveillance practice.

**Methods:**

The RSC’s pseudonymized primary care data, linked to hospital and other data, are held in the Oxford-RCGP Clinical Informatics Digital Hub, a Trusted Research Environment. We describe the RSC’s cohort profile as of September 2021, divided into a Primary Care Sentinel Cohort (PCSC)—collecting virological and serological specimens—and a larger group of syndromic surveillance general practices (SSGPs). We report changes to our sampling strategy that brings the RSC into alignment with European Centre for Disease Control guidance and then compare our cohort’s sociodemographic characteristics with Office for National Statistics data. We further describe influenza and COVID-19 vaccine coverage for the 2020-2021 season (week 40 of 2020 to week 39 of 2021), with the latter differentiated by vaccine brand. Finally, we report COVID-19–related outcomes in terms of hospitalization, intensive care unit (ICU) admission, and death.

**Results:**

As a response to COVID-19, the RSC grew from just over 500 PCSC practices in 2019 to 1879 practices in 2021 (PCSC, n=938; SSGP, n=1203). This represents 28.6% of English general practices and 30.59% (17,299,780/56,550,136) of the population. In the reporting period, the PCSC collected >8000 virology and >23,000 serology samples. The RSC population was broadly representative of the national population in terms of age, gender, ethnicity, National Health Service Region, socioeconomic status, obesity, and smoking habit. The RSC captured vaccine coverage data for influenza (n=5.4 million) and COVID-19, reporting dose one (n=11.9 million), two (n=11 million), and three (n=0.4 million) for the latter as well as brand-specific uptake data (AstraZeneca vaccine, n=11.6 million; Pfizer, n=10.8 million; and Moderna, n=0.7 million). The median (IQR) number of COVID-19 hospitalizations and ICU admissions was 1181 (559-1559) and 115 (50-174) per week, respectively.

**Conclusions:**

The RSC is broadly representative of the national population; its PCSC is geographically representative and its SSGPs are newly supporting UKHSA syndromic surveillance efforts. The network captures vaccine coverage and has expanded from reporting primary care attendances to providing data on onward hospital outcomes and deaths. The challenge remains to increase virological and serological sampling to monitor the effectiveness and waning of all vaccines available in a timely manner.

## Introduction

The emergence of SARS-CoV-2 and the resultant COVID-19 pandemic has reinforced the importance of continuous respiratory disease surveillance. However, processing routine health data comes with considerable challenges, requiring sophisticated digital infrastructure and data linkage to secondary data sources. The benefits afforded by such surveillance are contingent on data quality and timeliness.

The Oxford-Royal College of General Practitioners (RCGP) Research and Surveillance Centre (RSC) is now completing its 55th year of surveillance [1-3]. The University of Oxford’s Nuffield Department of Primary Care Health Sciences (NDPCHS) became the academic home for the RSC in 2019. As part of Oxford’s response to the emerging COVID-19 pandemic, the NDPCHS rapidly scaled up the RSC from its base of 500 practices in 2019 to over 1800 in 2021. What differentiates the RSC from other comparable disease surveillance networks is its integration of serology and virology sampling, and its close links between the RSC and network member practices.

The RSC works in partnership with the UK Health Security Agency (UKHSA), formerly Public Health England. RSC-led outputs include a surveillance report (Weekly Returns), published every week over the last 55 years, which increased to twice-weekly since the start of the pandemic, and an annual report [4]. Both are freely available online. In addition to these key outputs, there are a range of observatories providing contemporary national data; of note are our COVID-19 and mortality observatories. The RSC also provides weekly data for the European Centre for Disease Control (ECDC) on behalf of UKHSA.

RSC contributions to UKHSA intelligence include a large pseudonymized data set that enables vaccine effectiveness (eg, influenza vaccine) to be monitored. Of particular value is the differentiable data that enable subgroups of interest to be studied [5]. RSC data are stored securely in the Oxford-Royal College of General Practitioners Clinical Information Digital Hub (ORCHID) Trusted Research Environment (TRE). The ORCHID TRE offers multiple platforms for approved researchers to make use of. The surveillance platform (ORCHID-S) is the most developed, providing extended primary care surveillance. A trials platform (ORCHID-T) is in development, which will enable trial case identification and follow-up of consented patients, and an emergent epidemiology platform (ORCHID-E) that provides access to contemporary fully anonymized linked health data [6]. The ORCHID TRE facilitates the curation of clinical code sets and digital phenotypes used in computerized medical records (CMR) surveillance and research.

The objective of this study was to report the scale and representativeness of the RSC following a period of substantial growth, demonstrating the network’s value for monitoring vaccine uptake and a range of health outcomes, including hospitalization, intensive care unit (ICU) admission, and mortality. Specifically, we describe the representativeness of the RSC population at the end of the 2020-2021 surveillance season (September 30, 2021), its sentinel sampling, and data-linking procedures. This report also includes a description of the clinical informatics that underpins the network and our updated sentinel sampling criteria for the 2021-2022 season.

## Methods

### Surveillance Overview

The surveillance processes for using ORCHID TRE data were set out in 2020 [6]. Since that time, several additional features have been added: the growth in the size of the network; more frequent linkage to hospital and other data; the harnessing of a new daily data flow to support UKHSA’s real-time syndromic surveillance [7]; extending serosurveillance to evaluate COVID-19 immunity, with a particular focus on indications of waning immunity in vaccine risk groups; and adapting our practice liaison work to support member practices remotely.

Unless stated otherwise, our surveillance year starts on International Standards Organization (ISO) week 40 of 2020 and runs to the end of week 39 of the subsequent year. This approximates to the start of October 2020 to the end of September in the following year.

RSC practices are divided into two subcategories: the Primary Care Sentinel Cohort (PCSC) and syndromic surveillance general practices (SSGPs). There is overlap between these two groups of practices with 264 appearing in both.

### PCSC Characteristics

The PCSC is the longest established part of the RSC, with practices providing twice-weekly extracts of pseudonymized routine primary care data to UKHSA. Practice recruitment is nationally representative. A subset of the PCSC collects virology swabs and/or blood samples for virological and serological analysis. Only virological results are reported back to PCSC practices and the patient, although it must be stressed that this is not a diagnostic service. Practices are reimbursed for each virology and serology sample they submit for testing and code accurately. Both virology and serology samples are transported to UKHSA reference laboratories where they are analyzed and stored in dedicated biobanks for onward research.

From March 2022, the virology sampling criteria broadened to align with those of the ECDC. All patients presenting with symptoms consistent with influenza-like-illness (ILI), acute respiratory illness, or COVID-19 are now eligible for virological sampling (see Multimedia Appendix 1 for a visualization of overlapping symptoms that would confer eligibility for swabbing). Prior to this date, only those presenting with ILI, bronchitis or bronchiolitis (in those under 5 years of age), or COVID-19 were eligible. This new schema is included in our formal commissioning letter to our PCSC practices (see Multimedia Appendix 2).

Virology samples are obtained either in practice or within the patient’s own home via kits ordered online (supplied by Take A Test UK, an initiative of the nonprofit Saving Lives). This virological sampling is carried out in volunteer practices within the PCSC. Patients are eligible for swabbing if they present within 10 days of symptom onset (except within 14 days of a patient having received their live attenuated influenza vaccine). Samples are processed within the UKHSA’s Respiratory Virus Unit in Colindale, London. The UKHSA reference lab conducts an extended panel of tests for the presence of influenza A and B, respiratory syncytial virus A and B, COVID-19, metapneumoviruses, and (additionally this season) seasonal coronaviruses.

Serology samples are obtained opportunistically from volunteer patients attending for routine blood tests at sampling practices in the PCSC. These patients may be having blood tests as part of an acute illness or chronic disease management or prevention, leading to an overrepresentation of risk groups. These samples are primarily used to estimate population exposure to COVID-19; however, serological surveillance has also been successfully applied to monitoring levels of population exposure to influenza and diphtheria. This work also collects data on incidences of rare clotting events postvaccination via platelet factor 4 levels. Current serological tests for COVID-19–related antibodies include spike (S) antibodies (indicative of previous infection or vaccination) and nucleocapsid (N) antibodies (indicative of previous infection). Serology results are linked to RSC primary care records to compare SARS-CoV-2 S and N antibody results to existing patient health data and national seroprevalence studies [5]. Serology samples are managed by UKHSA’s Vaccine Evaluation Unit in Manchester.

### SSGP Characteristics

UKHSA’s national real-time syndromic surveillance service uses an existing combination of National Health Service (NHS) 111 calls and online assessments, ambulance dispatch calls, emergency department attendances, and general practitioner (GP) in- and out-of-hours consultations to monitor and identify trends that may indicate impending public health issues and events that might need intervention. SSGPs (all utilizing Egton Medical Information Systems [EMIS] Health clinical services) supply daily data to UKHSA to supplement and enhance the existing GP in-hours component of syndromic surveillance with a focus on respiratory disease. However, like the RSC’s Weekly Return, this will soon supply data on a wider range of diseases of interest [8]. This work is in its development and pilot stages.

### Data Sources

**Figure 1:**
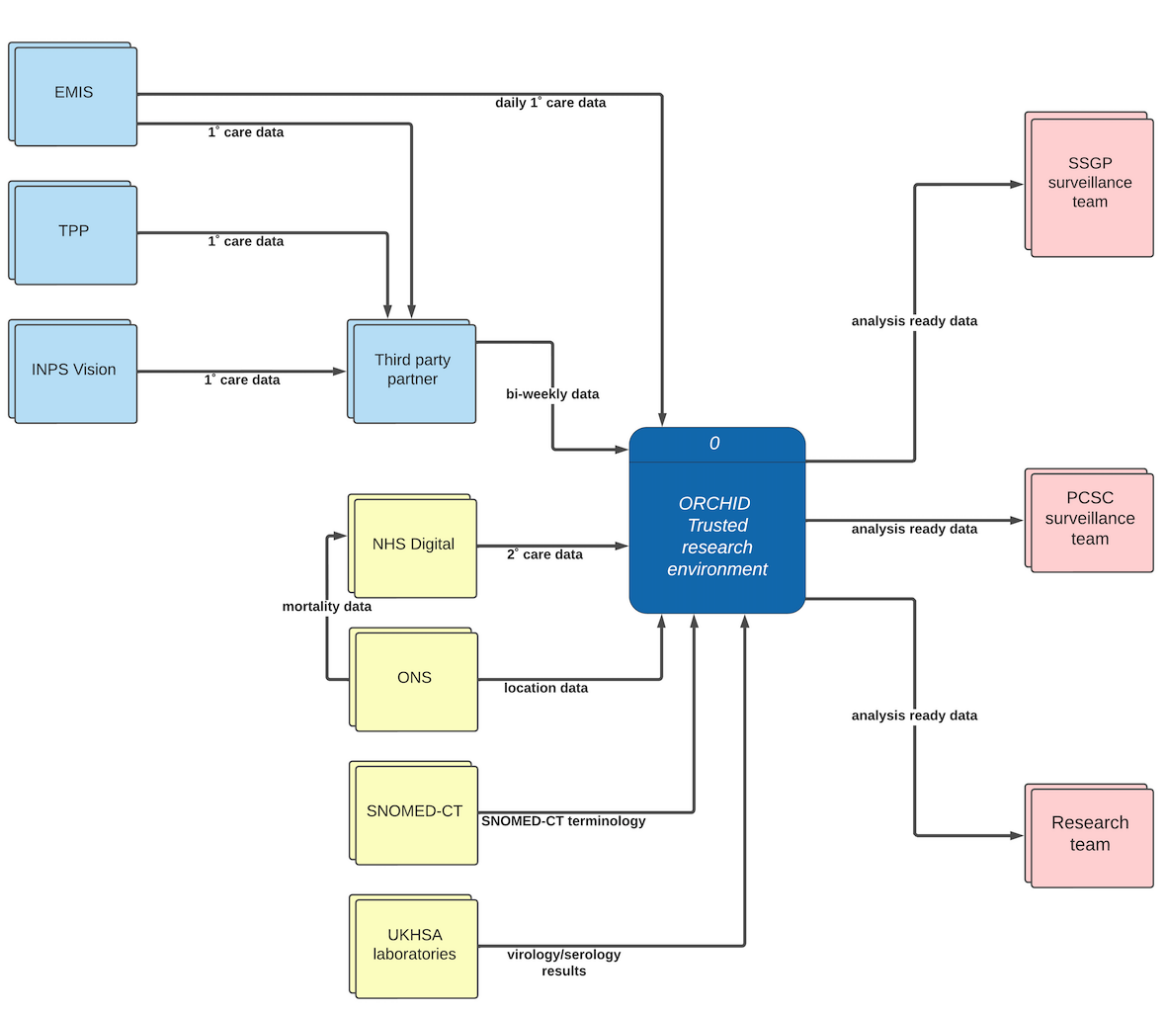
Data flow diagram for Oxford-Royal College of General Practitioners Clinical Information Digital Hub (ORCHID) Trusted Research Environment (TRE). Pale blue boxes represent the principal primary care data sources, pale yellow represents secondary data sources, and pale red represents data outputs. The dark blue box represents the data processing within the ORCHID TRE. Egton Medical Information Systems (EMIS) Health and The Phoenix Partnership (TPP) are primary care software services and act as data processors on behalf of National Health Service (NHS) primary care providers. INPS: In Practice Systems; ONS: Office for National Statistics; PCSC: Primary Care Sentinel Cohort; SNOMED-CT: Systematized Nomenclature of Medicine Clinical Terms; SSGP: syndromic surveillance general practices; UKHSA: UK Health Security Agency.

Figure 1 summarizes the health-related data sources used by the RSC and the constituent data flows of the ORCHID TRE.

The ORCHID TRE receives pseudonymized data from general practices (providing access to routine primary care data), NHS Digital (providing access to hospital, Office of National Statistics [ONS] death certificate data, and other national data sets), and UKHSA laboratories (who supply virology and serology results). Limitations on data usage are determined on a user-specific or project-specific basis. However, as set out in our data sharing agreement with practices, linking to other data sets is only permitted when done with the intention of enabling Health Surveillance, Quality Improvement, Research and/or Education (SQUIRE principles). The ORCHID TRE accepts data from any brand of CMR system. We currently receive data from EMIS, The Phoenix Partnership (TPP) System One, and In Practice Systems (INPS)-Vision. These data linkages are listed in full within Multimedia Appendix 3.

PCSC CMRs required for surveillance purposes are extracted twice weekly using a third-party company on behalf of the RSC. This process is managed by formal data sharing and service level agreements. Patient CMRs are pseudonymized using a nonreversible “hash” algorithm as close to the source as possible. Meanwhile, as stated, SSGP CMRs support UKHSA’s syndromic surveillance service by providing a direct feed of in-hours GP data to augment out-of-hours GP data, NHS 111, ambulance, and emergency department attendance data. Patients who decline to share their data are excluded from either extraction process.

### Scope of Data Collection

#### Data Capture

Data are captured at an individual pseudonymized level and patients’ general practice, which links a record to that practice’s relevant Lower Super Output Area (LSOA) level (minimum population of 1000, mean population of 1500), NHS administrative area, and NHS Region.

#### Sociodemographic Data

Sociodemographic data include age, gender, ethnicity, socioeconomic status (SES), NHS Region, rurality, smoking status, and obesity. Ethnicity is grouped into five categories: Asian, Black, white, mixed, and other. An ontology is used to maximize identification. SES is measured using the Index of Multiple Deprivation, a metric that is revealed by the LSOA level. NHS Region is defined using NHS Region mapping and is divided into seven areas: East of England, London, Midlands, North East and Yorkshire, North West, South East, and South West. Rurality is measured using ONS measures of population density, and divided into rural, town and city, and conurbation categories. Smoking status is categorized into nonsmoker, current smoker, and exsmoker. Obesity is categorized by BMI intervals (kg/m^2^), whereby <18.5 denotes underweight, 18.5-24.9 denotes normal weight, 25-29.9 denotes overweight, 30-34.9 denotes obese (obesity class I), 35-39.9 denotes obese class II, and 40 or above denotes morbid obesity (obesity class III).

#### Vaccine Uptake Data

The RSC provides data on vaccination uptake to its partners. Vaccine uptake data for COVID-19 are derived from RSC’s linkage to the National Immunisation Management Service (NIMS) [9]. Remaining vaccination data come directly from the primary care record.

#### Health Outcomes

For respiratory infectious disease surveillance and research, key health outcomes of interest are medically attended ILI/COVID-19 (qualified by admission to hospital, admission to ICU, and death). Relevant data are provided within CMRs or through linkages made with hospital data sets (eg, Health Episode Statistics) and ONS death registries via NHS Digital. These outcomes can be observed at a disease-specific level, and can be linked to reveal the entire patient journey and the variables that can predict excess risk.

#### Comorbidities and Other Variables

We provide a proxy variable to indicate consultation frequency and attendance. We also utilize the electronic frailty index for patients aged over 65 years and the Cambridge multimorbidity score within our data [10-12] (R Tsang, unpublished data, October 2022). Where possible, we provide a measure of household size to control for household bias when monitoring disease spread. This is done by applying a “household key” to RSC pseudonymized records. Here, groups of individuals are identified as living in a common address by flagging where records’ first line of an address and postcodes match. This matching is done at the point of data extraction from the GP system so that personal data are never revealed. This unique tool has been used in household transmission studies of acute gastroenteritis, influenza, and acute respiratory illnesses [13,14].

### Data Quality

The data quality within ORCHID TRE is underpinned by practice engagement, data capture, cleaning, aggregation, and analysis. The Practice Liaison Officers provide support and training, including on-site visits, for member practices. Other activities include personalized training, supply of sampling materials, webinars, and patient information, including information for patient participation groups. Practices are invited to access dedicated dashboards to monitor their sampling performance. We also publish overarching observatories with the goal of improving data quality among RSC members and provide specific support in data codification under our new *“Coding is caring”* initiative [15-17]. We have also started engaging with practice patient participation groups.

Our ontological mapping process recognizes that clinical concepts can be represented differently within a clinical terminology. To enable consistent and machine-readable identification of key outcomes and other variables [18], we use Systematized Nomenclature of Medicine Clinical Terms (SNOMED-CT) as the principal terminology to develop code sets [19]. A curated code set is a list of relevant clinical codes that best represent a specific clinical idea. All code sets are stored in our variable library. Hospital and death data are primarily coded using the International Classification of Disease version 10, with procedures recorded using the Office of Population Census and Surveys classification version 5.1 [20,21]. Treatments are codified in line with the descriptions and codes utilized by the Dictionary of Medicines and Devices [22]. All health conditions included in PCSC and SSGP surveillance, and their respective SNOMED-CT codes and our variable library numbers, are provided in Multimedia Appendix 4.

### Data Analysis

We accessed the secure ORCHID TRE using R version 4.2.0 to undertake all analyses [23]. We aggregated patient- and practice-level data to summarize characteristics of the network on October 8, 2021, including the number of practices that had agreed to share data, the number of participants actively supplying data, and key demographic variables. To establish network growth over the pandemic, we compared these data with historic records. Vaccine uptake figures were generated by aggregating primary care records linked to NIMS data. Hospitalization and ICU figures were created by aggregating primary care records linked to Hospital Episode Statistics data and mortality figures were generated using primary care data linked to ONS mortality figures.

### Ethical Considerations

We work within relevant legislation and research and information governance frameworks and are fully compliant with the University of Oxford’s ethical standards. The University is registered on the Information Commissioner’s Office Data Protection Register and is compliant with the Data Protection Act, General Data Protection Regulation, and other key data privacy and protection legislations. As required by NHS Data Security Standard 3 in the Caldicott 3 Review, all research members of NDPCHS are required to complete Data Security Awareness modules on an annual basis.

The legal basis for RSC surveillance is Regulation 3 (health protection) of the Health Service (Control of Patient Information) Regulations 2002, with some of our work with UKHSA falling under Regulation 5 (Health Promotion) [24,25]. Other nonsurveillance studies that use ORCHID TRE data require appropriate ethical approval. For low-risk epidemiological studies, this is through Oxford University Medical Sciences Interdivisional Research and Ethics Committee, whereas for trials or other prospective studies involving contact with patients, it is through the Integrated Research Approval Service [26]. All nonsurveillance studies also must be approved by the independent Primary Care Hosted Research Datasets Independent Scientific Committee. The RSC also meets NHS Digital’s stringent Data Security and Protection toolkit requirements. Finally, RSC activities are restricted to conform with the data sharing agreements with member practices, who, as stated, share data for SQUIRE purposes [6].

## Results

### Network Growth

The number of practices within the entire network prior to the emergence of SARS-CoV-2 (October 2019) was approximately 500 (all PCSC). The RSC network has grown substantially since that time. At the start of the reporting period (October 2020), however, the network included 1764 practices (879 PCSC practices, 1100 SSGPs); at its end (October 2021), it included 1879 practices (930 PCSC practices, 1203 SSGPs). By October 2021, there was an overlap of 262 practices supplying data to both groups. Table 1 shows the number of practices listed and samples collected as of ISO week 39 of 2021 and those recorded nationally (ONS data).

**Figure 2:**
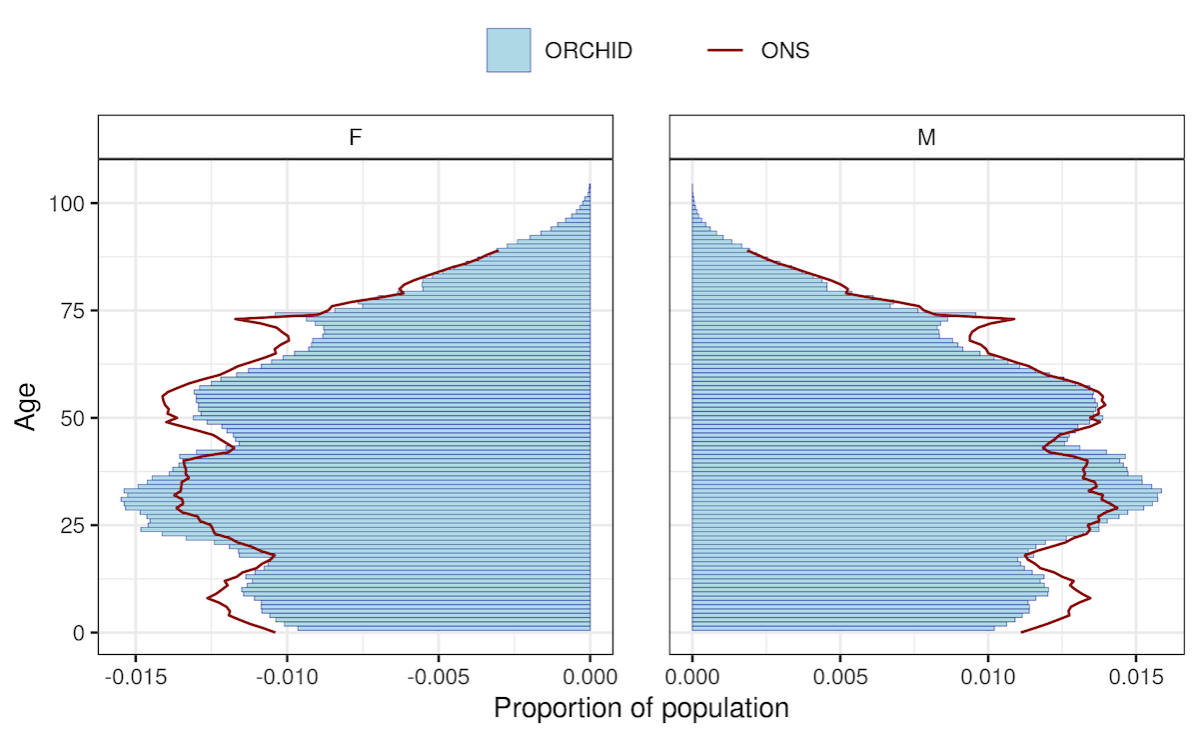
Age-sex pyramid of the Research and Surveillance Centre population on October 2021 compared to Office for National Statistics (ONS) estimates for 2019. F: female; M: male; ORCHID: Oxford-Royal College of General Practitioners Clinical Informatics Digital Hub.

Figure 2 presents a population pyramid of the age-sex profile of RSC data compared with the ONS report of the English national population’s age-sex profile. There was a higher proportion of younger working-age adults, aged 25 to 40 years, in the RSC population than the ONS standard. We describe the sociodemographic characteristics of the RSC compared to national population data in Table 2. This revealed higher levels of nonwhite ethnicity and active smoking among the RSC membership, and lower levels of females and population in the Eastern region in the RSC.

**Table 1:** Summary of Research and Surveillance Centre (RSC) practice and population sizes compared to national Office for National Statistics (ONS) data.

Data type	All RSC practices	PCSC^a^	SSGP^b^	National data (ONS)
General practices, n (%)^c^	1879 (28.63)	938 (14.29)	1203 (18.33)	6563 (100)
Registered list size, n (%)^c^	17,299,780 (30.59)	8,414,204 (14.88)	12,356,618 (21.9%)	56,550,136 (100)
Virology sampling practices, n	245	245	—^d^	—
Virology specimens, n	8049	8049	—	—
Serology sampling practices, n	220	220	—	—
Serology specimens, n	23,879	23,879	—	—

^a^PCSC: Primary Care Sentinel Cohort.

^b^SSGP: syndromic surveillance general practice.

^c^Percentage of national data (final column).

^d^Not applicable.

**Table 2:** Demographic characteristics of Research and Surveillance Centre (RSC) compared to Office of National Statistics (ONS) benchmark data.

Characteristics			All RSC surveillance practices^a,b^		Primary Care Sentinel Cohort^b^		Syndromic surveillance general practices^b^		English national data (ONS)
Age (years), median (IQR)^c^			39 (21-58)		39 (22-58)		38 (21-57)		40 (21-59)
Female, n (%)^c^			8,640,025 (49.9)		4,206,592 (50)		6,164,188 (49.9)		28,567,320 (50.5)
**Ethnicity, n (%)^c^**									
	White	11,347,155 (81.5)		5,585,337 (83.3)		8,108,670 (81)		47,417,500 (84.2)	
	Asian	1,390,875 (10)		622,009 (9.3)		1,022,726 (10.2)		4,661,000 (8.3)	
	Black	611,387 (4.4)		243,921 (3.6)		460,248 (4.6)		2,066,100 (3.7)	
	Mixed	310,100 (2.2)		142,891 (2.1)		224,780 (2.2)		1,086,600 (1.9)	
	Other	266,960 (1.9)		113,805 (1.7)		198,491 (2)		1,055,800 (1.9)	
**NHS^d^ Region, n (%)^c^**									
	South East	3,553,593 (20.5)		1,567,376 (18.6)		2,829,726 (22.9)		8,933,822 (15.8)	
	London	3,289,770 (19)		1,191,081 (14.2)		2,552,695 (20.7)		9,002,488 (15.9)	
	Midlands	2,791,986 (16.1)		1,171,549 (13.9)		2,076,374 (16.8)		10,658,558 (18.8)	
	North West	2,741,232 (15.8)		1,330,266 (15.8)		2,126,805 (17.2)		7,087,447 (12.5)	
	South West	2,124,626 (12.3)		1,523,323 (18.1)		1,164,724 (9.4)		5,665,799 (10)	
	North East Yorkshire	1,720,689 (9.9)		1,022,070 (12.1)		1,074,612 (8.7)		8,639,006 (15.3)	
	East of England	1,077,884 (6.2)		608,539 (7.2)		531,682 (4.3)		6,563,018 (11.6)	
**Index of multiple deprivation (IMD), n (%)^c^**									
	IMD1 (most deprived)	3,366,018 (19.5)		1,508,104 (17.9)		2,487,902 (20.1)		11,267,059 (20)	
	IMD2	3,483,363 (20.1)		1,648,030 (19.6)		2,480,510 (20.1)		11,576,973 (20.6)	
	IMD3	3,386,739 (19.6)		1,664,973 (19.8)		2,363,689 (19.1)		11,424,153 (20.3)	
	IMD4	3,408,200 (19.7)		1,764,757 (21)		2,398,673 (19.4)		11,117,694 (19.8)	
	IMD5 (least deprived)	3,652,825 (21.1)		1,826,114 (21.7)		2,625,435 (21.2)		10,901,082 (19.4)	
Morbid obesity, n (%)^e^			434,514 (2.5)		208,995 (2.5)		312,654 (2.5)		(3.2)^f^
**Smoking, n (%)^g^**									
	Active	2,304,974 (17.1)		1,106,166 (16.8)		1,659,899 (17.2)		4,897,952 (12.1)	
	Ex	3,236,336 (23.9)		1,608,336 (24.4)		2,297,591 (23.8)		10,645,963 (26.3)	
	Never	7,974,524 (59)		3,869,419 (58.8)		5,707,428 (59.1)		24,935,033 (61.6)	

^a^Data represent a cross-sectional view of the state of the RSC network on October 8, 2021.

^b^Represent the percentage of nonmissing data.

^c^ONS data based on 2019 estimates.

^d^NHS: National Health Service.

^e^ONS data based on 2020 estimates of morbid obesity in those aged over 16 years.

^f^Data based on a sample; therefore, only the percentage is provided.

^g^ONS data based on 2020 estimates of smoking status in those aged over 18 years.

### Geographical Profile

**Figure 3:**
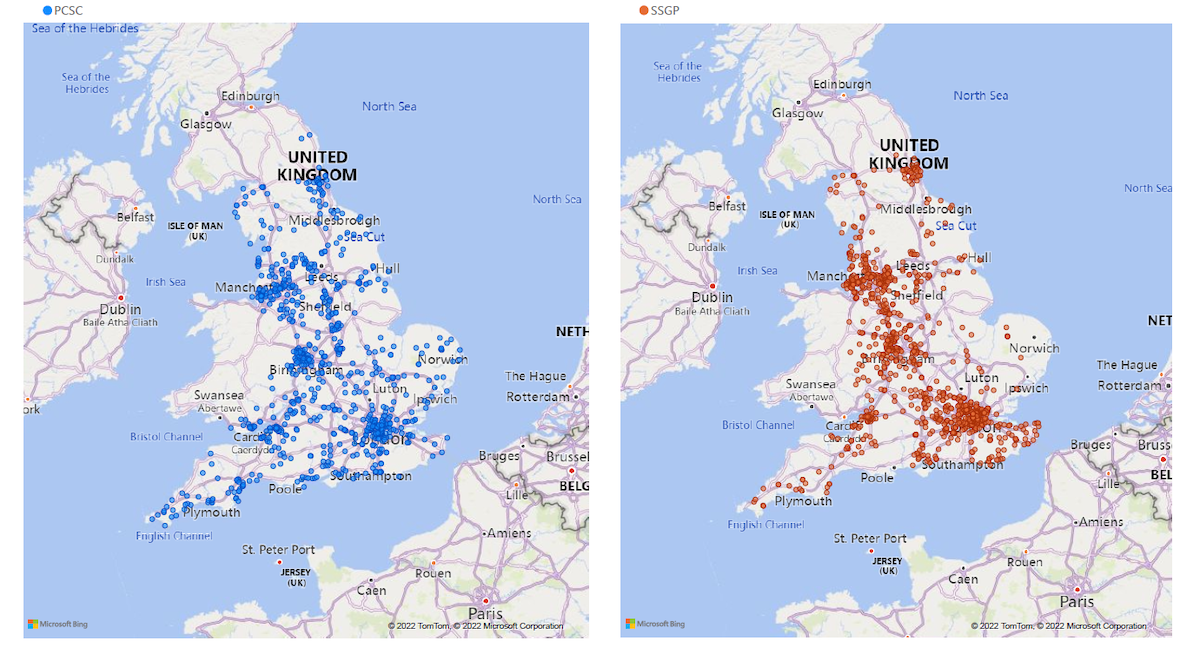
Geographical distribution of Primary Care Sentinel Cohort (PCSC) practices (left panel) and syndromic surveillance general practice (SSGP) practices (right panel).

The maps in Figure 3 demonstrate the national distribution of PCSC practices compared with SSGPs; note that these graphics omit the 264-practice overlap that exists between these two subcategories. The PCSC is recruited to be nationally represented, although has lower representation in the Eastern region, and the SSGPs are recruited from and follow the national distribution of the EMIS brand of the CMR system.

### Vaccine Uptake

**Figure 4:**
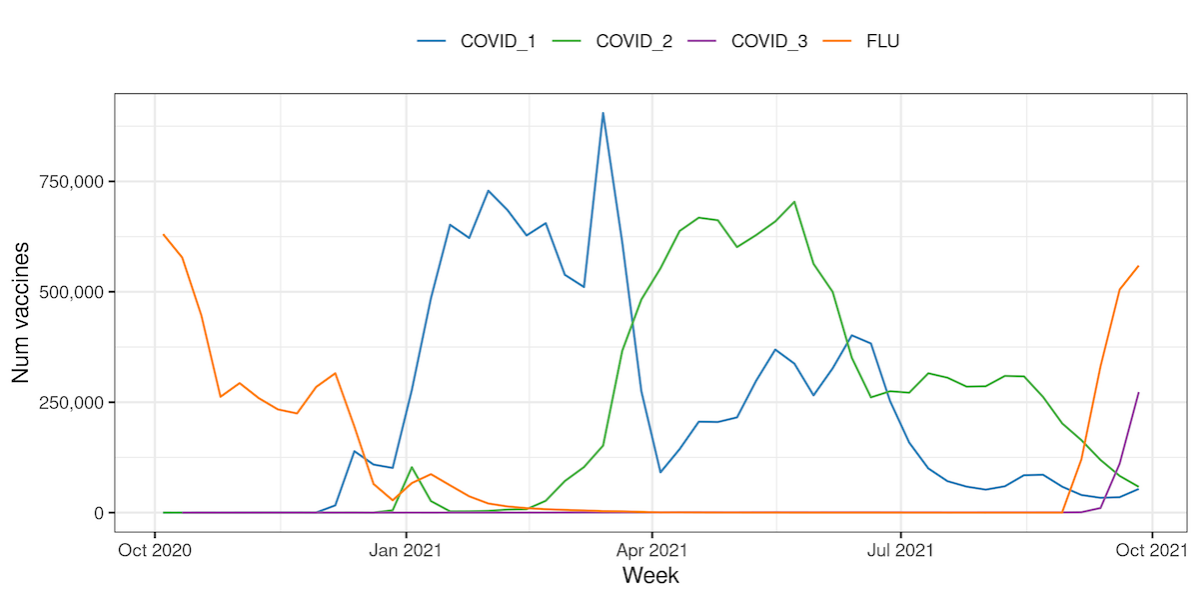
Absolute weekly vaccine uptake for seasonal influenza and COVID-19 doses over time. Data represent the entire Research and Surveillance Centre network population.

RSC data provide a profile of national vaccine uptake; RSC uptake data can be differentiated by vaccine type, brand, and batch in addition to the demographics of those who have been vaccinated (Figure 4 and Multimedia Appendix 5). Across the RSC, there were 11,897,180 first, 10,992,049 second, and 397,986 third/booster doses of COVID-19 vaccine (a total of 23,287,215), and 5,387,169 doses of influenza vaccine administrations recorded between October 2020 and the end of September 2021. The brands of vaccine administered over this period were AstraZeneca (n=11,632,841), Pfizer (n=10,849,114), and Moderna (n=694,696).

### Health Outcomes

**Figure 5:**
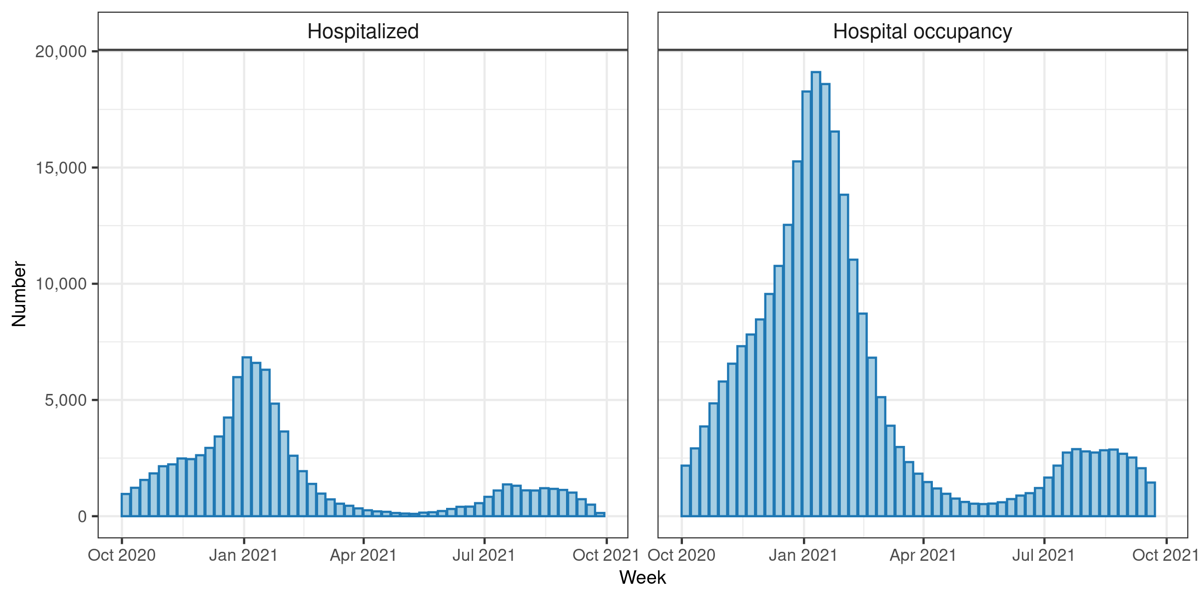
COVID-19 hospitalization data in the RSC network population, calculated using RSC data linked to Hospital Episode Statistics (HES) data. Left panel: Number of hospitalisations per week due to COVID-19. Right panel: Weekly hospital bed occupancy. An admission was defined as spending at least 1 night in hospital.

**Figure 6:**
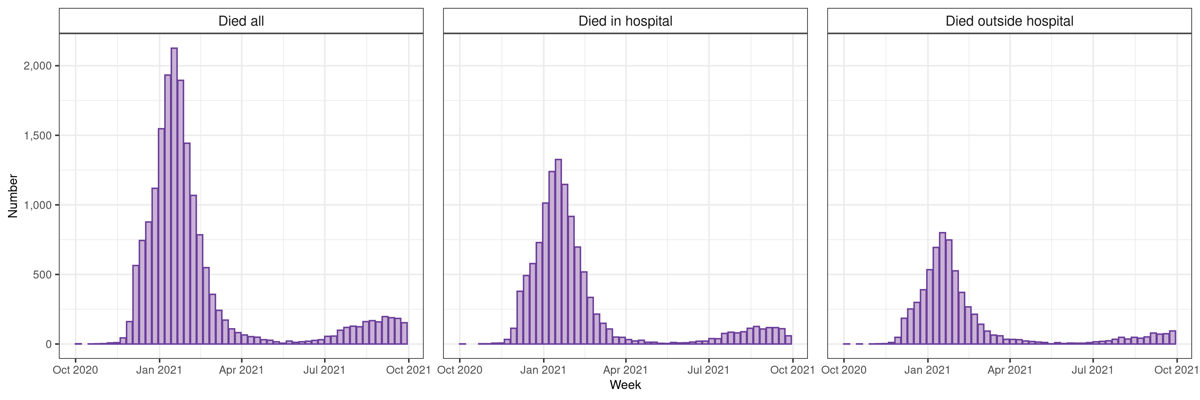
COVID-19 death data in the Research and Surveillance Centre (RSC) network population, calculated using RSC data linked to Office of National Statistics death data and COVID-19 Hospitalizations in England Surveillance System data. Most deaths occurring between October and December 2020 are not shown due to missing data. Left panel: All deaths. Middle panel: In-hospital deaths. Right panel: Out of hospital deaths.

Figure 5 reports the COVID-19–specific hospitalization and hospital occupancy; Figure 6 reports the death data, separating hospital and community mortality; and Multimedia Appendix 6 summarizes the ICU admission and ICU occupancy rates. Over this period, COVID-19 admissions varied from 104 to 6835 per week (median 1181, IQR 559-1559). Bed occupancy over this period varied from 557 to 19,110 per week (median 3284, IQR 1512-7443). For the ICU, the equivalent data were admissions varying from 6 to 842 per week (median 115, IQR 50-174), with ICU occupancy varying from 103 to 2451 per week (median 504.5, IQR 213.5-813.0).

### Practice Visits and Patient Participation

Restrictions due to COVID-19 have limited our ability to conduct visits to practices. However, over this period, there were 25 virtual and 5 in-person visits. There were monthly newsletters, 5 training webinars, and the completion of 483 practice material requests to sampling practices. We have updated and provided practices with bespoke dashboards about influenza vaccination, their data quality, and—for sampling practices—numbers of virology swabs completed and serology samples taken. We provided practice members with new COVID-19 case and mortality observatories. We have commenced a pilot of direct engagement with five patient participation groups of sampling practices to test whether giving more direct health and surveillance information to patients and the public will enhance sampling. Eight practices left the network during the 2020-2021 season.

## Discussion

### Main Findings

This summary cohort profile demonstrates how the RSC provides contemporaneous data about disease patterns, emerging illnesses, circulating viral infections and their variants, population immunity, and vaccine coverage. The RSC has changed and broadened its virology sampling criteria during the specified reporting period.

The RSC has also grown significantly in recent years by adding SSGPs to its PCSC; historically, the RSC processed data from approximately 1 million patients and we now have data from 17.3 million registered people, including nearly 12 million people who have had at least one COVID-19 vaccine dose. We are now starting a pilot of direct engagement with patients and the public. The innovation to informatics over the reporting period included consistently curated code sets of variables, improved data management (with more data linkages, including to hospital and death data), and improved data availability for subsequent analyses.

### Implications of the Findings

The capability of the RSC has grown, embedding serology sampling alongside virology. However, there is more to be done in this area, particularly as national virological testing for COVID-19 is decommissioned and UKHSA may increasingly look toward sentinel sampling to compensate for this loss. We hope our broadened sampling criteria will result in the collection of a larger number of virology samples, as will building out our direct contacts with patients and the public.

### Comparison With the Literature

RSC surveillance is comparable to that conducted internationally, and similar approaches to monitoring disease have been used in developed health systems [27]. These include the Canadian Primary Care Sentinel Surveillance Network, the US Influenza-like Illness Surveillance Program, the National Respiratory and Enteric Virus Surveillance System (also US-based), and the *Sentinelles* network in France [28-31].

### Strengths and Limitations

The functions and outputs of the RSC are aligned with the priorities and ambitions specified by UKHSA in their Information and Intelligence Working Group “Public Health Surveillance: Towards a Public Health Surveillance Strategy for England” [32]. Effective surveillance is categorized via its ongoing nature, its timeliness, and the measures of population or group health status it provides against historical or geographical baselines. All of these are observable within the RSC results reported here. The wider objectives of a robust surveillance system include:

- Monitoring changes in infectious agents
- Providing early warning of seasonal disease activity and future emerging threats through an enhanced national GP syndromic surveillance system
- Identifying high-risk populations or areas to target interventions
- Evaluating the effectiveness of preventative health and health control measures
- Supporting health planning and the allocation of appropriate resources within the health care system
- Providing an archive of disease activity (or biological samples) for future reference and research.

The quality of surveillance is contingent on the extent to which its findings are generalizable to its underlying population. ORCHID TRE data now approximate to national data for age, gender, ethnicity, SES, rurality, smoking status, and obesity. This enables nuanced analyses for how certain demographics and individuals with protected characteristics are at higher risk for experiencing health inequalities and adverse outcomes. There remains scope to improve the representativeness of the RSC. The practices that volunteer are typically larger than average, are not equally distributed between regions, and are, overall, from slightly less deprived areas. There may also be other undetected forms of bias in our membership. We need to recruit more practices in the eastern region and target recruitment into the PCSC to geographically balance membership. However, pressures on primary care make recruitment and retention of practices challenging [33,34].

The UKHSA syndromic surveillance service also utilizes anonymized CMR data from an external feed of TPP primary care data; we will need to work closely with these providers to ensure overall national representativeness of this offering and the standardization of coding underpinning surveillance indicators. Signals that emerge through syndromic surveillance data can be validated against wider ORCHID TRE data and its opportunities for broader data linkage. Integrating SSGP data into the existing UKHSA syndromic surveillance program will also increase the application of ORCHID TRE data in a multihazard public health response, including surveillance of nonrespiratory infectious diseases (eg, gastrointestinal pathogens), environmental impacts (eg, heat waves), chemical incidents (eg, health impacts of industrial fires), and mass gatherings (eg, 2022 Birmingham Commonwealth Games).

We are aware of attendance bias in those who provide the RSC with samples. In virology sampling, it is well-established that some families and population groups attend more frequently with respiratory illnesses than is nationally representative [35,36]. Serology sampling is also seen to be biased toward those attending more regularly for blood tests, especially older people with chronic conditions [37].

### Conclusions

This cohort profile describes the RSC’s capabilities for conducting disease surveillance and vaccine effectiveness studies, and provides a guide to network components and capabilities. While there remains scope for improvement, the RSC is now stronger and larger than at any time in its 55-year history, particularly in terms of sampling performance. Its 2020-2021 end of surveillance year registered population was 17.3 million, accounting for 31% of the English national population. This population was seen to collectively receive over 23 million COVID-19 vaccination doses. We move through the 2021 to 2022 season with revised sampling criteria and, for the first time, daily data contributions to the UKHSA real-time syndromic surveillance service.

## Appendix

Multimedia Appendix 1 Research and Surveillance Centre (RSC) Primary Care Sentinel Cohort (PCSC) Virology sampling criteria. ARI: acute respiratory illness; ILI: influenza-like illness; LRTI: lower respiratory tract infection; URTI: upper respiratory tract infection.

Multimedia Appendix 2 Commissioning letter to Primary Care Sentinel Cohort (PCSC) practices.

Multimedia Appendix 3 Oxford-Royal College of General Practitioners Clinical Information Digital Hub (ORCHID) linkage to other data sets.

Multimedia Appendix 4 Systematized Nomenclature of Medicine Clinical Terms (SNOMED-CT) code sets used for surveillance in the Oxford-Royal College of General Practitioners Clinical Information Digital Hub (ORCHID) Trusted Research Environment (TRE) variable library.

Multimedia Appendix 5 Absolute weekly COVID-19 vaccine uptake over time differentiated by vaccine type.

Multimedia Appendix 6 COVID-19 intensive care unit (ICU) data in the Research and Surveillance Centre (RSC) network population.

## Abbreviations

CMR: computerized medical record
ECDC: European Centre for Disease Control
EMIS: Egton Medical Information System
GP: general practitioner
HPRU: Health Protection Research Unit
ICU: intensive care unit
ILI: influenza-like illness
INPS: In Practice Systems
ISO: International Standards Organization
LSOA: Lower Level Super Output Area
NDPCHS: Nuffield Department of Primary Care Health Sciences
NHS: National Health Service
NIHR: National Institute for Health Research
NIMS: National Immunisation Management Service
ONS: Office of National Statistics
ORCHID: Oxford-Royal College of General Practitioners Clinical Informatics Digital Hub
PCSC: Primary Care Sentinel Cohort
RGCP: Royal College of General Practitioners
RSC: Research and Surveillance Centre
SES: socioeconomic status
SNOMED-CT: Systematized Nomenclature of Medicine Clinical Terms
SQUIRE: Surveillance, Quality Improvement, Research, Education
SSGP: Syndromic Surveillance General Practices
TPP: The Phoenix Partnership
TRE: Trusted Research Environment
UKHSA: United Kingdom Health Security Agency
