# Supplementary figures and images for: Representativeness, Vaccination Uptake, and COVID-19 Clinical Outcomes 2020-2021 in the UK Oxford-Royal College of General Practitioners Research and Surveillance Network: Cohort Profile Summary
Source: JMIR Public Health Surveill. 2022 Dec 19;8(12):e39141. doi: 10.2196/39141 (PMC9770023; doi:10.2196/39141)

**Multimedia Appendix 1: RSC PCSC Virology sampling criteria**


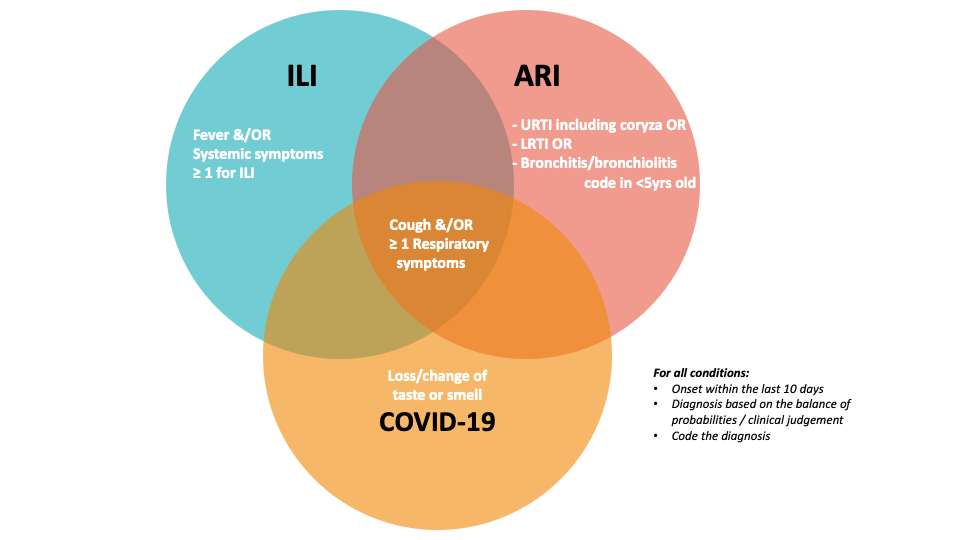

Supplement: Multimedia Appendix 1 [file publichealth_v8i12e39141_app1.docx]
