# Supplementary material for: Representativeness, Vaccination Uptake, and COVID-19 Clinical Outcomes 2020-2021 in the UK Oxford-Royal College of General Practitioners Research and Surveillance Network: Cohort Profile Summary
Source: JMIR Public Health Surveill. 2022 Dec 19;8(12):e39141. doi: 10.2196/39141 (PMC9770023; doi:10.2196/39141)
Supplement: Multimedia Appendix 2 [file publichealth_v8i12e39141_app2.pdf]

UK Health Security Agency | RCGP Royal College of General Practitioners Research & Surveillance Centre | University of Oxford | ORCHID Oxford–Royal College of General Practitioners Clinical Informatics Digital Hub

Respiratory Virus & Vaccine Effectiveness Surveillance Programme 2022/2023

September 2022

Dear Surveillance Practice,

Thank you for being a member of the Oxford-Royal College of General Practitioners Clinical Informatics Digital Hub (ORCHID), which is part of the Oxford-Royal College of General Practitioners (RCGP) Research and Surveillance Centre (RSC). We are especially grateful for your contribution to our virology swabbing and serology sampling surveillance.

This year, we commence our 56th season of disease surveillance in collaboration with the UK Health Security Agency (UKHSA). Since the early 1990s, a subset of Oxford-RCGP RSC network practices have volunteered to conduct virology swabbing during the winter months. In 2018 and 2019, serology sampling was successfully instituted.

Virology and serology sampling is used to monitor SARS-CoV-2, measure background population immunity to infectious diseases, report on vaccine effectiveness, and support ethically approved research. Virology sampling is also used to monitor other very important respiratory conditions, such as influenza (flu), and respiratory syncytial virus (RSV). Since 2020, virology and serology sampling has been all-year round. The Oxford-RCGP RSC also monitors vaccination uptake for flu and COVID-19.

The value of your contribution is immense. Your swabs help to signal early detection of outbreaks e.g. for flu or RSV. Data on the types of strains or variants circulating is provided to the World Health Organisation (WHO) to inform important decisions on which vaccines to use for the winter season. Blood samples and vaccine uptake data is used by UKHSA for analysis on vaccine effectiveness and waning, which is particularly important in clinical risk groups.

We greatly appreciate your continuing collaboration.

Yours faithfully,

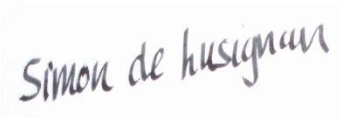
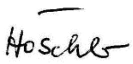

Professor Simon de Lusignan
Director
RCGP RSC
simon.delusignan@phc.ox.ac.uk

Katja Hoschler
Deputy Head
Respiratory Virus Unit, VRD, UKHSA Colindale
katja.Hoschler@ukhsa.gov.uk

Introduction

State of the Art Surveillance
You are one of over 1,900 general practices who already volunteer to share pseudonymised (de-identified) patient data with us.

The RSC has been the primary source for disease surveillance in England for over 60 years. We work closely with the UK Health Security Agency (UKHSA), and its predecessor organisations, to monitor diseases around the country, confirm cases of influenza and other respiratory viruses, provide vaccine effectiveness estimates, and characterise vulnerable populations. Practice data is used to produce a weekly Communicable and Respiratory Disease Report, reporting on 37 monitoring conditions, which is available at RCGP Public Health Data. Data reported by UKHSA is available at National flu and COVID-19 surveillance reports.

We also support other areas of surveillance with UKHSA. These include syndromic surveillance (a broader form of surveillance also looking at non-communicable diseases), more intensive gastroenteritis surveillance, and responsive surveillance based on data requirements that arise in UKHSA from time to time e.g. paediatric hepatitis, monkey pox and diphtheria.

ORCHID Data Sharing
Full details regarding the legal basis for our work are outlined in our ORCHID Data Sharing Agreement (DSA) and ORCHID Privacy Notices. The legal basis for our surveillance activity is The Health Service (Control of Patient Information) Regulations 2002 – Regulation 3.

Data is only used for surveillance, quality improvement, research and education (SQuIRE) purposes. Patients who have opted out of sharing their GP records (e.g. by contacting their GP or through the NHS national data opt out service) will not have their data processed.

"Coding is Caring"
Disease surveillance is for the benefit of patients, but the data relies entirely on high quality data being coded into the patient's computerised medical record (CMR) (not as free text or scanned documents/attachments). Hence our motto "Coding is Caring". Our key areas are:

- Problem coding with episode typing
- Vaccine exposure – especially flu vaccines (further details below)
- Virology swabbing and serology sampling (if participating)
- Virology results (if participating)

"Sampling is Informing"
Around 300 of our network practices also volunteer to do virology swabbing and serology sampling. These samples get linked to coded data from the patient's computerised medical record. Being able to link sample results with patient data in this way is what makes our network particularly unique and valuable.

Virology swabs help us to identify which viruses are circulating. Of particular interest is influenza (flu), respiratory syncytial virus (RSV), and SARS-CoV-2. Usually, sampling would take place during the winter season (from around September to March). However, since 2020, this

has continued all-year round. Blood samples are also used to formally measure vaccine effectiveness, and provide information about background levels of population immunity to infectious diseases. This information is critical for National Health Service (NHS) planning and provides the World Health Organisation (WHO) key data for global vaccine strain selection meetings, which occur twice a year. Hence our motto "Sampling is Informing".

Improving Data Quality and Usefulness

Vaccine effectiveness estimates rely heavily on sample information being completed fully. Please be sure that all information requested on laboratory forms and sample tubes is completed. Otherwise, samples may have to be excluded, and practices may not be paid for their hard work. Analysis of swabs collected in the 2019/20 season indicated that around 12% of swabs were excluded from vaccine effectiveness analysis due to missing data. This was an improvement on 25% of swabs excluded in the 2017/2018 season.

My Practice Dashboard

By joining the network, every practice is issued a unique 'dashboard key' to access My Practice Dashboard. The dashboard uses practice data to provide practice level informatics to support practices should they wish to use it e.g. to support QOF targets, improve episode typing, problem coding, track virology or serology sampling progress, or flu vaccine uptake (and potential practice earnings). These dashboards show progress against other practices in your region, as well as, the network as a whole.

We also have a number of network-wide observatories available on the ORCHID website, for example:

- Serology Observatory
- COVID-19 Observatory
- Social Prescribing Observatory
- Workload Observatory

Practice Support

We have a dedicated team of Practice Liaison Officers (PLOs). Please contact them if you have any queries: practiceenquiries@phc.ox.ac.uk. Keep an eye out for our weekly updates, monthly newsletters, and webinars. The PLO team is also available for practice visits (in-person or online) to provide training and advice.

Please share

Please share this information with your entire practice and Patient Participation Group (PPG). Please also include surveillance activity in information you share about your practice with the Care Quality Commission.

Coding is Caring

Key Data Requirements:

1. **Code conditions as a 'problem':** Wherever possible, please code a diagnosis as a 'problem' in the patient's computerised medical record using diagnostic SNOMED terms. This should be based on the balance of probabilities/clinical judgement. E.g. If

the likely diagnosis is influenza, code as ‘influenza-like illness’ (or influenza if you are certain). Avoid coding symptoms e.g. ‘cough’ or vague concepts like ‘viral illness’. Especially important are our monitored respiratory and infectious conditions.

2. **Code episode type:** For effective disease surveillance, it is important to understand whether conditions are new or on-going. Please code the problem as first, new, on-going, or review (depending on your clinical system e.g. EMIS, SystmOne TPP, or if using Ardens).

Episode typing algorithm

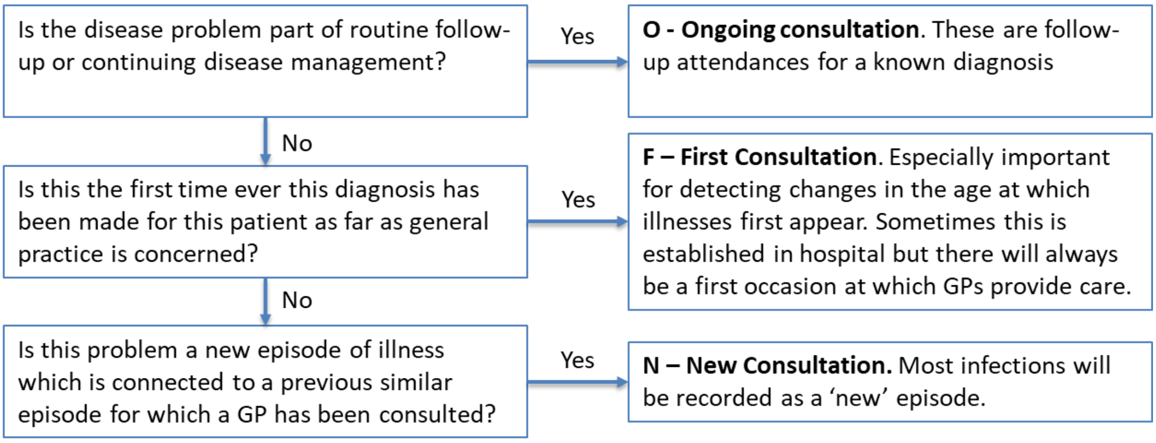

3. **Code vaccination, especially brand and batch for flu:** It is really important to record this information since there is more diversity in types of flu vaccines available to different age groups. Out-of-practice vaccination is a challenge, so please try to record as much information as possible about out-of-practice vaccination, including brand of vaccine and batch number. Without complete and correct coding flu vaccine effectiveness measures are much less reliable. COVID-19 vaccination is largely automated, so not as much of a problem but please also record COVID-19 vaccination if not in the clinical records (e.g. conducted overseas).
4. **Code virology and serology has been taken:** It’s important to code into the patient’s computerised medical record that a virology swab or serology sample has been taken.
5. **Code virology swabbing results:** For vaccine effectiveness estimates, it is vital that virology results are coded into the patient’s computerised medical record, even if negative. Please do not just scan documents in as we cannot extract documents attached to patient’s medical record). Please include the date of collection to the results.

Sampling is Informing

- **Verbal consent only:** For virology swabbing and serology sampling, patients only need to give verbal consent. Written consent is not required as stipulated in The Health Service (Control of Patient Information) Regulations 2002 – Regulation 3.

- **Patient Information Sheet:** The Patient Information Sheet for virology and serology sampling is available online should you wish to hand these to patients or text them a link.
- **Swab and sample across all age bands:** In general we ask that practices swab and sample across all age bands. However, for virology surveillance we want more swabs from the under 5s, under 18s and over 65s.
- **Further information:** Further information is available at our All Year General Virology and Serology Surveillance webpage. This includes our latest Respiratory Virus and Vaccine Effectiveness Protocol.
- **Additional pilot studies:** Occasionally, we may reach out to practices to volunteer for pilot studies to enhance our surveillance sampling. These may include trialling new point-of-care-testing machines (POCT), or using EMIS Recruit technology. Further details under 'Serology Sampling'.

Virology Swabbing

Virology swabbing provides critical information on which respiratory viruses are circulating and is used to measure vaccine effectiveness. Practices are reimbursed £12.50 per swab.

Who to swab

Since March 2022, we have expanded our swabbing criteria. Practices should be on the look-out and swab suspected cases of:

- Influenza-like illness (ILI), or
- Acute respiratory infection (ARI), or
- Suspected COVID-19

ARI includes lower respiratory tract infection (LRTI) and upper respiratory tract infection (URTI). In the under 5 year olds, please be on the look-out for cases of bronchitis/bronchiolitis – this is especially important for monitoring cases of RSV.

Virology Swabbing Criteria

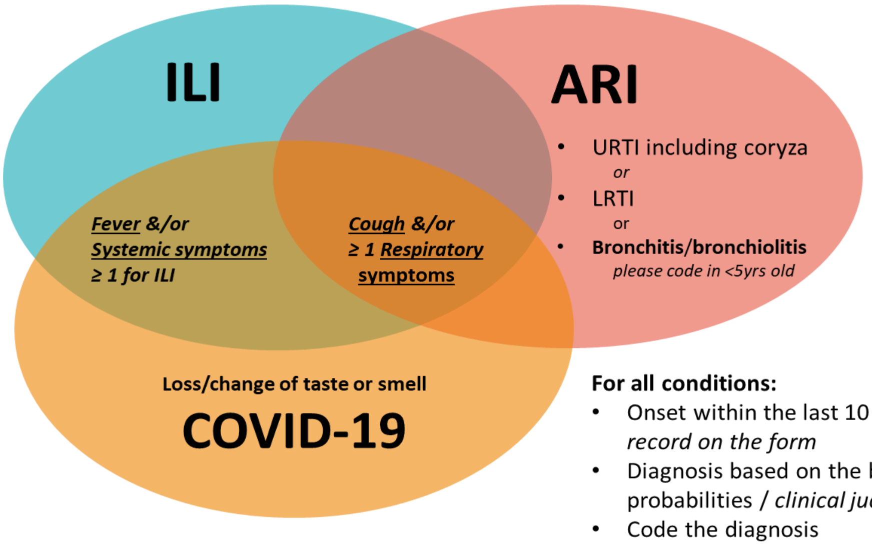

For all conditions:

- Onset within the last 10 days – *please record on the form*
- Diagnosis based on the balance of probabilities / *clinical judgement*
- Code the diagnosis

Systemic symptoms include: Fever/chills, headache, malaise, muscle/body aches, weakness, fatigue/lethargy, diarrhoea, nausea, vomiting.
Respiratory symptoms include: cough, shortness of breath, difficulty breathing, sore throat, sneezing, runny/stuffy nose, wheezing.

Targets

- **Swab across all age bands***
- **Take 20 swabs per week**

Suggested swabbing targets by age band

**Age Band**	**Number of swabs per week**
Under 5s	5 swabs
Under 18s	5 swabs
18-65	5 swabs
65+	5 swabs
**Total**	**20 swabs**

*If possible swab across different age bands, especially the under 18s, under 5s, or adults over 65. This is important because:

a) Under 18s and over 65s are administered different vaccine types.
b) The under 18s are given the live attenuated influenza vaccine (LAIV) and we are investigating the effectiveness of intranasal vaccination.
c) RSV causes a significant level of morbidity and mortality, particularly in young infants. There are at least five candidate immunisations at phase 2 or 3 clinical trial. There is a need to establish RSV disease burden in the UK, particularly in younger children and the elderly (e.g. those with COPD) to inform optimal future use of these new vaccines and to provide a baseline for subsequent impact studies stage.

Method

Practices have 3 options for taking virology swabs:

1. **Clinician takes a swab using in-practice kits provided by the PLO team**: In-practice kits can be ordered using the Material Requests portal.
2. **Clinician gives patient the in-practice kit to take home**: Please be sure to complete the virology lab form with the patient, especially date of sample collection, date of onset, and patient's NHS number.
3. **Clinician directs patient to TakeATestUK website**: Practices must provide patients with the practice code (and NHS number if possible) for the patient to order a self-swab kit to their home. Patients will receive results via text for flu and COVID-19. All other results will have to be disseminated by the practice.

Practice Workflow for Virology Swabbing

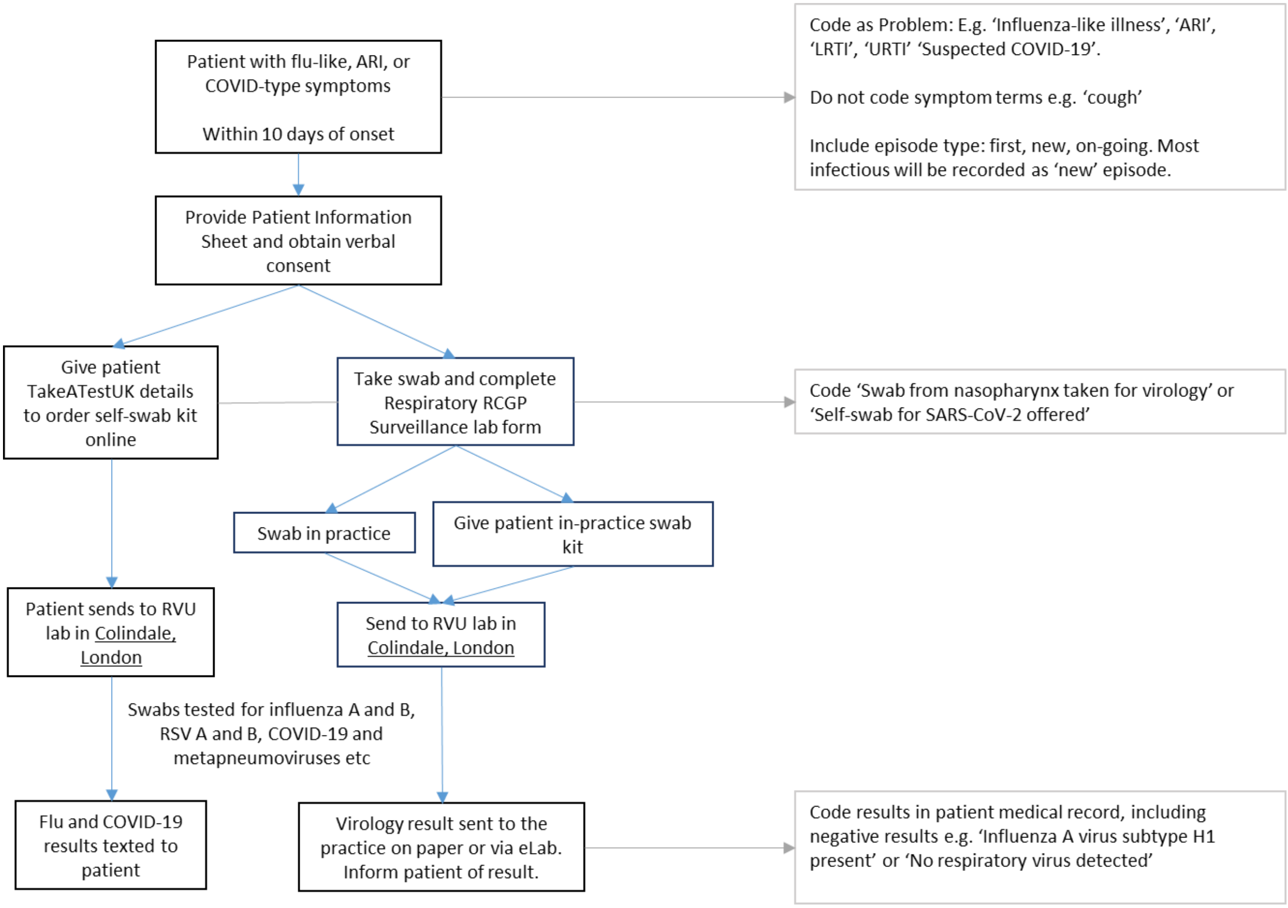

Instructions for In-Practice Kits

- Order in-practice kits from the PLO team using the Material Requests portal. You must reference your UKHSA lab code.
- Check the bottom of the box before emailing about potentially missing items.
- When taking a swab, it is important to abrade the mucosal surfaces of the throat and nose with the cotton buds and not simply dip the bud into nasal mucus or oral saliva.
- Ensure that every field is completed on the virology lab form, especially: date of sample collection, date of onset, and patient details (especially the patient's NHS number, age and sex). Please be sure to complete the lab form if you are handing the in-practice kit to the patient to take home.
- Clearly label the swab tube so they can be matched with the lab form. Otherwise the sample may get discarded. The following information must be included: NHS number, name, date of birth, sex and date of collection.
- Remember to post the lab form with the sample.
- Make sure samples are well packed. If they leak during transit they will get discarded.

- Post samples on the same day. If this is not possible, samples should be kept in a fridge at +4C and sent to us at the earliest opportunity.

Virology kit and lab form

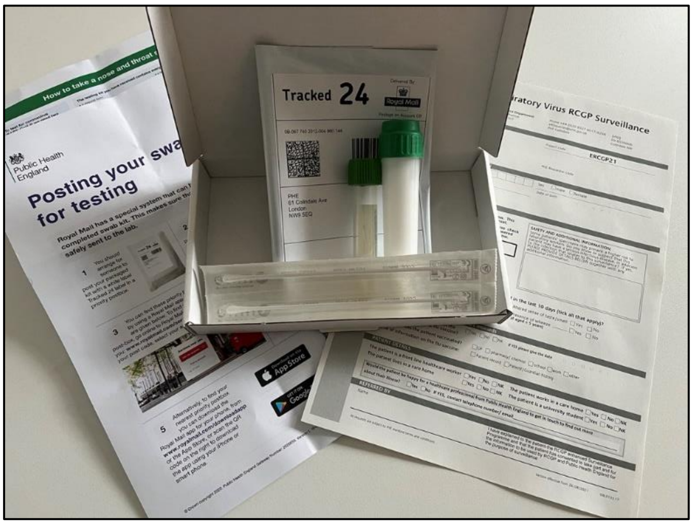

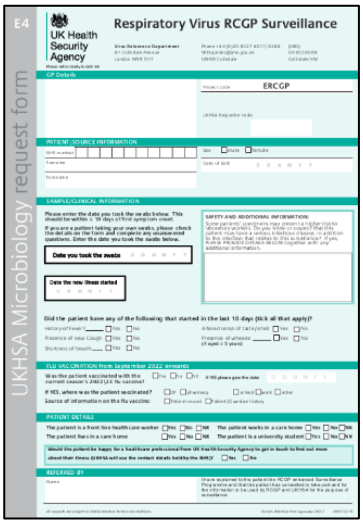

Instructions for TakeATestUK

- Make sure the PLO team have registered the practice to the TakeATestUK service.
- Ensure patients are eligible. Patients must still fulfil the swabbing criteria e.g. symptomatic within 10 days of onset.
- Check that the patient has a mobile phone they can use to receive results via SMS.
- Give the patient the practice ODS code.
- Direct them to https://takeatestuk.com.
- Further guidance can be found at UK Health Security Agency Flu and COVID 19 Survey.

TakeATestUK webpage

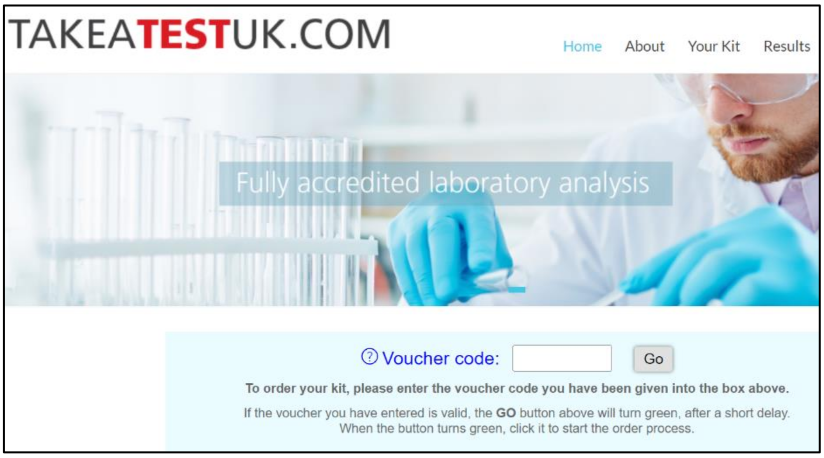

Laboratory Results

In the 2022/23 season, we will continue to test virology swabs by PCR for: SARS-CoV-2, influenza (A, B, H1, H3), respiratory syncytial virus (A and B), hMPV, seasonal coronaviruses (229E, OC43, NL63, HKU1), rhinovirus, enterovirus, and adenovirus.

Whether using in-practice kits or TakeATestUK, all results will be sent back to the practice on paper in the post or electronically via eLab (as a PDF). eLab is the electronic messaging service provided by UKHSA. Patients using TakeATestUK will only receive results for flu and COVID-19. If the result is positive e.g. for RSV, the practice will need to notify the patient.

Influenza positive samples may be selected for further detailed analysis using influenza genome sequencing, and for some also selected for antigenic characterisation of the virus. These data are used to compare how similar the currently circulating influenza viruses are to the strains included in seasonal influenza vaccines, and to monitor for changes in circulating influenza viruses. The information obtained is an important component of the UK influenza surveillance.

Results Coding

Regardless of whether the practice receives results by post or via eLab, practices must still manually code all results into the patient's computerised medical record, even if negative.

Please record the data of collection of the results wherever possible. This information, along with the recommended SNOMED CTs for virology lab results can also be found on the lab result issued by UKHSA.

**Currently recommended SNOMED Clinical Terms for Virology Lab Results**
SARS-CoV-2 (severe acute respiratory syndrome coronavirus 2) detected
SARS-CoV-2 (severe acute respiratory syndrome coronavirus 2) not detected
Influenza A virus subtype H1 present
Influenza A virus subtype H3 present
Influenza B virus present
Influenza A virus subtype H1N1 detected
Respiratory syncytial virus A present
Respiratory syncytial virus B present
Human Metapneumovirus present
Human Adenovirus present
Human Coronavirus 229E
Human Coronavirus HKU1
Human Coronavirus NL63
Human Coronavirus OC43
Human Enterovirus present
Human Rhinovirus present
**No respiratory virus detected**

Critical Information for UKHSA

- Only take swabs from patients presenting within 10 days of symptom onset.
- It is extremely important to record the 1) date of sample collection, and 2) date of symptom onset on the virology lab form.
- The virology lab form will ask if the patient received the flu vaccine this year (2022/23), date of vaccination and place of vaccination (GP/pharmacy/school/workplace/other). If possible, also code this information in to the patient's computerised medical record.
- Swabbing should be undertaken regardless of flu or COVID-19 vaccination status.
- However, no swabs should be taken from patients who have received a Live Attenuated Influenza Vaccine (LAIV) in the last 14 days.
- Do not swab more than two patients from a single household or care home per week.

Tips

1. Identify which staff members will interact with symptomatic patients. This could be the duty doctor or a nurse, in person or on the phone. We suggest taking two swabs per rotation (4 per day).
2. Set up your AccuRX or other patient messaging service so patients can be sent and SMS with the Patient Information Sheet or TakeATestUK instructions.
3. Distribute swabbing kits in rooms where symptomatic patients are seen.
4. Check expiry dates and use up kit. This will help cut down on unused kits going to waste.
5. Consider adding surveillance to the agenda at a Patient Participation Group meeting.
6. Use My Practice Dashboard to view your progress and to view what viruses are circulating in the community, or how much money could be made through sampling or flu vaccinations.

Serology Sampling

Serology samples provide information about background levels of population immunity. Blood samples are tested with a number of assays to look at levels of exposure to COVID-19 infection and responses to vaccination. From time to time samples may also be used for testing antibody levels for other communicable diseases which may be circulating in the community for the effectiveness of associated vaccines. No results are given to patients or their GP.

Practices are reimbursed:

- £5.50 for over 18s
- £11 for 8-17s
- £30 for under 8s

Who to Sample

Anyone attending a pre-scheduled blood test. Simply take an additional bottle of blood using the kits provided.

Targets

- **Sample across all age bands***
- **Sample across all regions****
- **5-20 samples per week**

*We are particularly interested in higher risk populations e.g. older age groups, and those from ethnic minority backgrounds. This season we would also like more samples from younger people (teenagers and younger).

**We also welcome more samples from practices in the North West, London, Midlands, and the East of England.

Practice Workflow for Serology Sampling

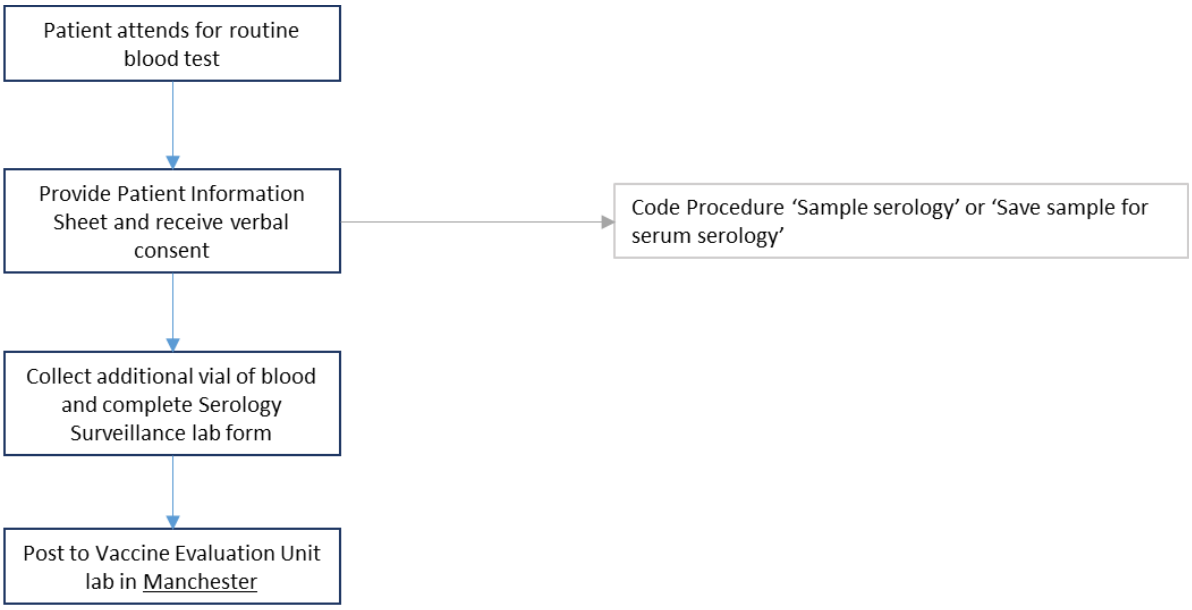

Samples used for population-wide monitoring of flu and COVID-19 immunity. Patients and practices do not receive results.

Instructions for Serology Kits

- Order in-practice kits from the PLO team using the Material Requests portal. We supply BD Vaccutainer. Practices can use their own Monovette supplies. In this case, please notify the PLO team that you only require the pre-paid boxes/envelopes, not the sample bottles.

- All fields on the UKHSA serology lab must be completed.
- Make sure the sample tube contains the following information: Practice Name, Patient Name, NHS Number, DOB, Sex, and Date of Collection.
- Ensure the blood bottle is completely filled e.g. 10 ml if supplied the red top, 8.5 ml if supplied the yellow top. Insufficient samples are discarded.
- Inset the filled blood tube into the transport container with the rubber stopper facing out. Do not force the rubber stopper into the transport tube.

Serology kit and lab form

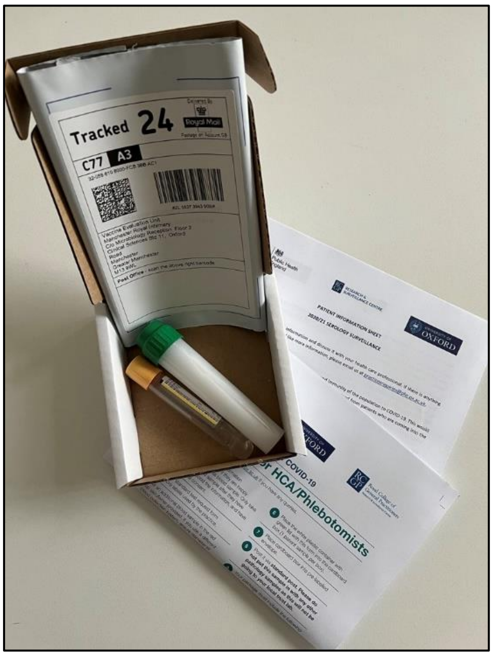

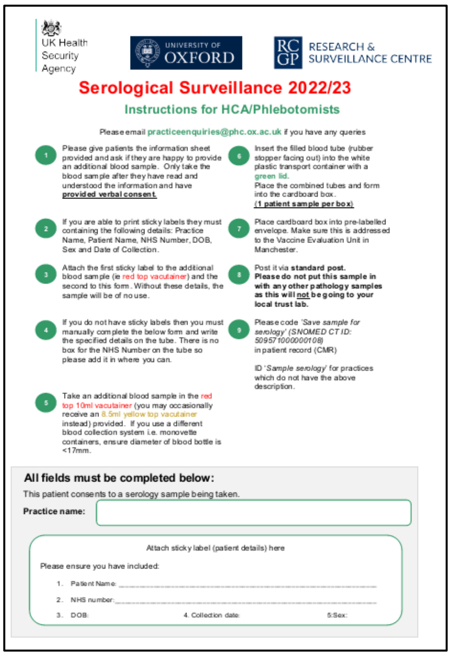

UK Health Security Agency | University of Oxford | RCGP Research & Surveillance Centre

Serological Surveillance 2022/23

Instructions for HCA/Phlebotomists

Please email practiceenquiries@phc.ox.ac.uk if you have any queries

1. Please give patients the information sheet provided and ask if they are happy to provide an additional blood sample. Only take the blood sample after they have read and understood the information and have **provided verbal consent.**
2. If you are able to print sticky labels they must containing the following details: Practice Name, Patient Name, NHS Number, DOB, Sex and Date of Collection.
3. Attach the first sticky label to the additional blood sample (ie red top vacutainer) and the second to this form. Without these details, the sample will be of no use.
4. If you do not have sticky labels then you must manually complete the below form and write the specified details on the tube. There is no box for the NHS Number on the tube so please add it in where you can.
5. Take an additional blood sample in the red top 10ml vacutainer (you may occasionally receive an 8.5ml yellow top vacutainer instead) provided. If you use a different blood collection system i.e. monovette containers, ensure diameter of blood bottle is <17mm.
6. Insert the filled blood tube (rubber stopper facing out) into the white plastic transport container with a green lid. Place the combined tubes and form into the cardboard box. **(1 patient sample per box)**
7. Place cardboard box into pre-labelled envelope. Make sure this is addressed to the Vaccine Evaluation Unit in Manchester.
8. Post it via **standard post. Please do not put this sample in with any other pathology samples as this will not be going to your local trust lab.**
9. Please code *'Save sample for serology' (SNOMED CT ID: 509571000000108)* in patient record (CMR)

   ID *'Sample serology'* for practices which do not have the above description.

All fields must be completed below:

This patient consents to a serology sample being taken.

Practice name:

Attach sticky label (patient details) here

Please ensure you have included:

1. Patient Name: ____________________
2. NHS number: ____________________
3. DOB: 4. Collection date: 5:Sex:

Correct and incorrect way of inserting blood sample into sample tube

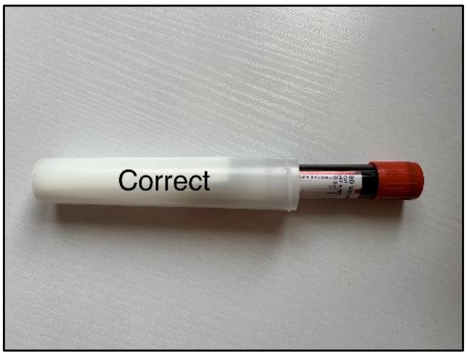

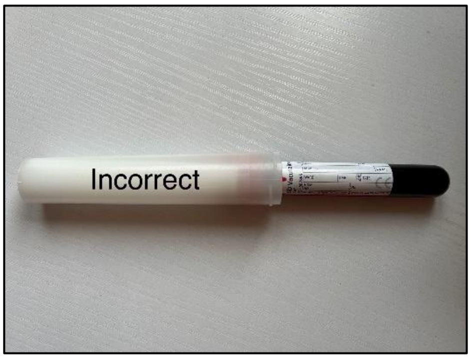

Results

No results come back to the practice or patient from serology testing

Tips

1. Ask the reception team to review upcoming blood test appointments for the next 24-48 hours and send an SMS to patients with a mobile number containing information

about serology surveillance and a link to the Patient Information Sheet. This will save time during the appointment. In some cases it has increased uptake as patients often request/remind phlebotomists to take the extra sample for research.
2. Make sure there are serology kits available in every HCA room and nominate a phlebotomist to monitor serology kit levels and order kits early to avoid running out.
3. Have extra paper copies of the Patient Information Sheet to hand.
4. If possible, print extra patient ID stickers to affix to the serology lab form and serology bottle.
5. Place coding reminders on computers in HCA rooms. Coding can be done manually or with a template e.g. if using EMIS, add a shortcut on F12 key. Send regular reminders about the importance of coding.
6. Update the HCA team quarterly e.g. number of serology samples taken, income from serology sampling.
7. Make it work with your practice workflow. The HCA team should feel it adds value to their work rather than be a source of stress. There may be days where sampling is more challenging e.g. if the practice is short-staffed.

Additional Pilot Study

We would like to use EMIS recruit to automatically flag patients (who are having blood collected for clinical reasons), whose samples may be particularly valuable for serological surveillance, such as repeat samples in immunocompromised patients to monitor response to COVID and flu vaccines. If you use EMIS Recruit or plan to start this winter and are interested in contributing please contact meredith.leston@linacre.ox.ac.uk

If you have any questions, please contact the Practice Liaison Officer (PLO) team at: practiceenquiries@phc.ox.ac.uk.
