# Supplementary material for: Representativeness, Vaccination Uptake, and COVID-19 Clinical Outcomes 2020-2021 in the UK Oxford-Royal College of General Practitioners Research and Surveillance Network: Cohort Profile Summary
Source: JMIR Public Health Surveill. 2022 Dec 19;8(12):e39141. doi: 10.2196/39141 (PMC9770023; doi:10.2196/39141)

**Multimedia Appendix 3: ORCHID linkage to other datasets**

Linkage to other data sets to support surveillance and research; individual data sets may have limitations on their use to specific projects and users.


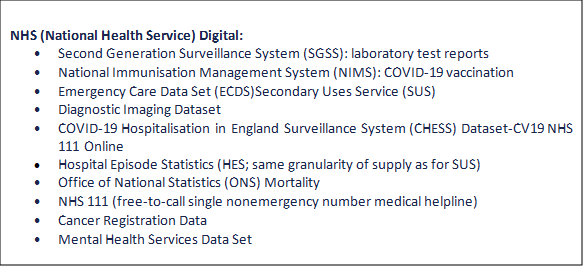

Supplement: Multimedia Appendix 3 [file publichealth_v8i12e39141_app3.docx]
