# Supplementary material for: Representativeness, Vaccination Uptake, and COVID-19 Clinical Outcomes 2020-2021 in the UK Oxford-Royal College of General Practitioners Research and Surveillance Network: Cohort Profile Summary
Source: JMIR Public Health Surveill. 2022 Dec 19;8(12):e39141. doi: 10.2196/39141 (PMC9770023; doi:10.2196/39141)
Supplement: Multimedia Appendix 4 [file publichealth_v8i12e39141_app4.docx]

**Multimedia appendix 4**

SNOMED-CT code sets in the ORCHID TRE variable library provided by the PCSC for surveillance and weekly reports

| **Condition group** | **Code set** | **Library No** |
| --- | --- | --- |
| **Respiratory** | Allergic Rhinitis | 1057 |
|  | Asthma | 1008 |
|  | Bronchitis | 1058 |
|  | Common Cold | 1069 |
|  | COVID-19 – clinically confirmed | 4857 |
|  | COVID 19 – laboratory confirmed | 4856 |
|  | Influenza-like illness | 1020 |
|  | Laryngitis and Tracheitis | 1003 |
|  | Lower Respiratory Tract Infections | 1055 |
|  | Otitis Media Acute | 1018 |
|  | Strep Throat and Peritonsillar Abscess | 1005 |
|  | Pleurisy | 1036 |
|  | Pneumonia and Pneumonitis | 1052 |
|  | Respiratory System Diseases | 1022 |
|  | Sinusitis | 1054 |
|  | Symptoms involving Respiratory and Chest | 1028 |
|  | Tonsillitis and acute Pharyngitis | 1063 |
|  | Upper Respiratory Tract Infections | 1007 |
|  | Whooping Cough | 1044 |
| **Non-respiratory** | Bullous Dermatoses | 1067 |
|  | Chickenpox | 1011 |
|  | Conjunctival Disorders | 1035 |
|  | Herpes Simplex | 1037 |
|  | Herpes Zoster | 1060 |
|  | Impetigo | 1031 |
|  | Infectious Mononucleosis | 1061 |
|  | Intestinal Infectious Diseases | 1006 |
|  | Measles | 1068 |
|  | Meningitis and Encephalitis | 1045 |
|  | Mumps | 1030 |
|  | Non-infective Enteritis and Colitis | 1048 |
|  | Peripheral Nervous Disease | 1066 |
|  | Rubella | 1021 |
|  | Skin and Subcutaneous Tissue Infections | 1062 |
|  | Symptoms involving musculoskeletal | 1001 |
|  | Symptoms involving Skin and Integument Tissues | 1038 |
|  | Urinary Tract Infections | 1046 |
|  | Viral Hepatitis | 1056 |

SNOMED-CT code sets in the ORCHID TRE variable library provided by the SSGP for syndromic surveillance

| **Condition group** | **Code set** | **Variable No** |
| --- | --- | --- |
| **Respiratory** | Acute tonsillitis/pharyngitis | 1063 |
|  | Allergic rhinitis | 1057 |
|  | Asthma | 1008 |
|  | Bronchiolitis | 3109 |
|  | Bronchitis | 1058 |
|  | COVID-19 – clinically confirmed | 4857 |
|  | COVID 19 – laboratory confirmed | 4856 |
|  | COVID-19 - suspected | 4855 |
|  | Influenza like illness | 1020 |
|  | Lower Respiratory Tract Infection | 1055 |
|  | Pertussis | 1044 |
|  | Upper Respiratory Tract Infection | 1007 |
| **Non-respiratory** |  |  |
|  | Gastroenteritis | 4858 |
|  | Heat stroke | 4860 |
|  | Herpes zoster | 1060 |
|  | Mumps | 1030 |
|  | Presumed infectious diarrhoea | 4859 |
|  | Scarlet fever | 3111 |
|  | Vomiting | 4583 |
