# Supplementary material for: Representativeness, Vaccination Uptake, and COVID-19 Clinical Outcomes 2020-2021 in the UK Oxford-Royal College of General Practitioners Research and Surveillance Network: Cohort Profile Summary
Source: JMIR Public Health Surveill. 2022 Dec 19;8(12):e39141. doi: 10.2196/39141 (PMC9770023; doi:10.2196/39141)

**Multimedia Appendix 6: COVID-19 intensive care unit (ICU) data in the RSC network population**

COVID-19 intensive care unit (ICU) data in the RSC network population, calculated using RSC data linked to Hospital Episode Statistics (HES) data. Where date of discharge from ICU was missing this was imputed. **Left panel:** Number of ICU admissions per week due to COVID-19. **Right panel**: Weekly ICU bed occupancy. An ICU admission was defined as any length of time spent in ICU, including where individuals did not spend a night in the ICU.


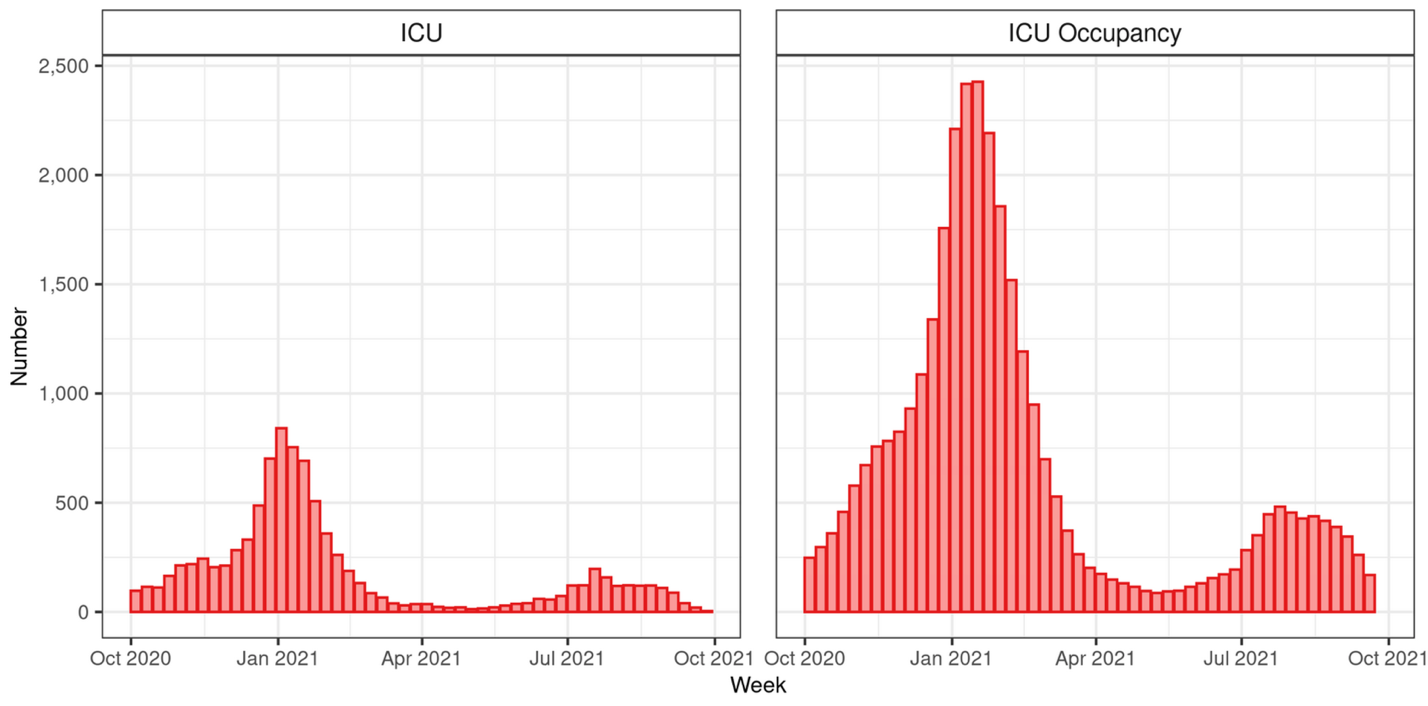

Supplement: Multimedia Appendix 6 [file publichealth_v8i12e39141_app6.docx]
